# Care coordination in PMAQ-AB: an Item Response Theory-based analysis

**DOI:** 10.11606/S1518-8787.2017051007024

**Published:** 2017-09-11

**Authors:** Miriam Francisco de Souza, Alaneir de Fatima dos Santos, Ilka Afonso Reis, Marcos Antônio da Cunha Santos, Alzira de Oliveira Jorge, Antônio Tomaz Gonzaga da Matta Machado, Eli Iola Gurgel Andrade, Mariangela Leal Cherchiglia

**Affiliations:** I Programa de Pós-Graduação em Saúde Pública. Faculdade de Medicina. Universidade Federal de Minas Gerais. Belo Horizonte, MG, Brasil; II Departamento de Medicina Preventiva e Social. Faculdade de Medicina. Universidade Federal de Minas Gerais. Belo Horizonte, MG, Brasil; III Departamento de Estatística. Instituto de Ciências Exatas. Universidade Federal de Minas Gerais. Belo Horizonte, MG, Brasil; IV Programa de Pós-Graduação em Saúde Pública. Departamento de Medicina Preventiva e Social. Faculdade de Medicina. Universidade Federal de Minas Gerais. Belo Horizonte, MG, Brasil

**Keywords:** Primary Health Care, Health Services Accessibility, Health Services Evaluation, Efficiency, Organizational, Quality Assurance, Health Care

## Abstract

**OBJECTIVE:**

Analyze the quality of the National Program for Primary Care Access and Quality Improvement variables to evaluate the coordination of primary care.

**METHODS:**

A cross-sectional study based on data from 17,202 primary care teams that participated in the National Program for Primary Care Access and Quality Improvement in 2012. Based on the Item Response Theory, Samejima’s Gradual Response Model was used to estimate the score related to the level of coordination. The Cronbach’s alpha and Spearman’ coefficients and the point-biserial correlation were used to analyze the internal consistency and the correlation between the items and between the items and the total score. We evaluated the assumptions of unidimensionality and local independence of the items. Cloud-type word charts aided in the interpretation of coordination levels.

**RESULTS:**

The Program items with the greatest discrimination in coordination level were: telephone/Internet existence, institutional communication flows, and matrix support actions. The specialists’ contact frequency with the primary care and integrated electronic medical record required a greater level of coordination among the teams. The Cronbach’ alpha was 0.8018. The institutional communication flows and telephone/Internet items had a higher correlation with the total score. Coordination scores ranged from -2.67 (minimum) to 2.83 (maximum). More communication, information exchange, matrix support, health care in the territory and the domicile had a significant influence on the levels of coordination.

**CONCLUSIONS:**

The ability to provide information and the frequency of contact among professionals are important elements for a comprehensive, continuous and high-quality care.

## RESUMO

**OBJETIVO:**

Analisar a qualidade das variáveis do Programa de Melhoria do Acesso e da Qualidade da Atenção Básica para avaliar a coordenação na atenção básica do cuidado.

**MÉTODOS:**

Estudo transversal baseado em dados de 17.202 equipes de atenção básica que participaram do Programa de Melhoria do Acesso e da Qualidade da Atenção Básica em 2012. Baseado na Teoria de Resposta ao Item, o Modelo de Resposta Gradual de Samejima foi utilizado para estimação do escore relacionado ao nível de coordenação. Os coeficientes alfa de Cronbach, Spearman e ponto bisserial foram utilizados para análise da consistência interna e da correlação entre os itens e de itens com o escore total. Foram avaliadas as suposições de unidimensionalidade e de independência local dos itens. Gráficos do tipo nuvem de palavras auxiliaram na interpretação dos níveis de coordenação.

**RESULTADOS:**

Os itens do Programa com maior discriminação do nível de coordenação foram: existência de telefone/internet, fluxos institucionais de comunicação e ações de apoio matricial. A frequência de contato de especialistas com a atenção básica e prontuário eletrônico integrado exigiram maior nível de coordenação das equipes. O coeficiente alfa de Cronbach total 0,8018. Os itens fluxos institucional de comunicação e telefone/internet tiveram maior correlação com o escore total. Os escores de coordenação variaram entre -2,67 (mínimo) e 2,83 (máximo). Maior grau de comunicação, troca de informações, apoio matricial, cuidado no território e domicílio tiveram peso relevante nos níveis de coordenação.

**CONCLUSÕES:**

A capacidade de disponibilizar a informação e a frequência de contato entre os profissionais são elementos importantes para o cuidado abrangente, contínuo e de qualidade.

## INTRODUCTION

Primary Health Care (APS) as the care coordinator has been a subject of discussion in several countries. Health systems recognize that health services must respond to health needs and demands in a comprehensive, coherent and cost-effective manner^2^.

Changes in the epidemiological and demographic profile of the world population, especially with an aging population and an increase in the prevalence of chronic diseases, requiring more complex and coordinated care among different services^13^. Although the literature indicates positive responses in systems that focus on the strength of APS, changes and investments are indispensable to guarantee this coordinated care^6^.

The challenges for APS to coordinate care reflect the need to revise resource allocation patterns in APS; the network management, focused on the individual and geared towards chronic conditions; the provision and transfer of information through integrated information technologies; and the credibility of the APS, which needs society’s support and trust^11^.

The search for care quality and continuity has exposed several coordination strategies, although these strategies do not always achieve the desired result. This may be related to the lack of a consensual definition and, in part, to a lack of clarity about the forms of intervention and measurement of care coordination^22^. However, it can be understood as an organizational mechanism that guarantees continued and integral care, an essential attribute of APS. The continuity of care and problem recognition by the individuals involved in the care are important elements to evaluate the coordination^21^.

The growing interest in health systems coordinated by APS has helped the increase in coordination processes analysis. The Agency for Healthcare Research and Quality published the Care Coordination Measures Atlas, updated in 2014, which provides evaluation methodologies^a^. Issues such as organizational mechanisms for improving coordination, strategies, and performance of health systems are present in this context.

A recent systematic review^20^ indicates the availability of the information is the aspect most often analyzed. Most studies consider the patient’s perspective, with only 27% of instruments addressing the perspective of health professionals. In these, accountability and the establishment of goals for care were the most frequent aspects^20^.

In Brazil, the National Program for Primary Care Access and Quality Improvement (PMAQ-AB), created the Ministry of Health (MH) in 2011, is a strategy to induce increased APS access and improved quality^b^. The Ministry of Health (MH) reinforces APS and poses the challenge of establishing the Family Health Strategy (ESF) as the center of the Health Care Networks (RAS) in health care ordering and coordination. The information resulting from the analysis of the actions of primary care teams who act in different scenarios can contribute to the improvement of care by indicating potentialities, fragilities, and challenges standing in the way of the health care actions effectiveness.

The APS’s capacity to coordinate care is much discussed in Brazilian studies. There is research on strategies to strengthen the APS^2^ ^,^ ^6^ ^,^ ^12^, the position that APS assumes in the RAS and the coordination attribute^1^ ^,^ ^5^ ^,^ ^8^. These studies are relevant to indicate the changes and investments required by APS. Rodrigues et al.^16^ pointed out as a challenge the need for studies with more robust methodological delineations and valid evidence of the APS’s capacity to coordinate RAS.

In PMAQ-AB, primary care teams receive quality certification according to their performance in monitoring agreed health indicators and in checking a set of quality standards. This set of quality standards can be analyzed according to the Item Response Theory, relating a certain quality standard to the probability of the team’s response or behavior. Thus, our variable of interest is the care coordination level, which, although not directly observable, can be estimated from the answers provided by the primary care teams in the Program^14^.

In view of the above, the present study aimed to analyze the quality of the National Primary Care Access and Quality Improvement Program variables to evaluate primary care as a care coordinator in Brazil.

## METHODS

Cross-sectional study based on the use of the Item Response Theory (IRT) using the data collected in 2012, during the external evaluation phase of PMAQ-AB, in a partnership between MH and research and learning institutions throughout the country. About 17,202 primary care teams (50% of the teams registered in 2011) participated in this study, on a voluntary basis, and they had joined the PMAQ-AB.

The external evaluation is the third phase of the PMAQ-AB, complementary to three other phases: the first, formalization of the adhesion and contracting of municipalities and teams, followed by the development phase; and the last one, the recontextualization phase, is a cyclical and systematic process. The questionnaire used had the objective of ascertaining the conditions of access and quality of all participating municipalities and teams. It consists of three modules and, in the present study, only Module II was used, which consists of an interview with a professional about the primary care team’s work process.

The variables selection when using the Item Response Theory was based on domains and conceptions for care coordination verified in the literature. Initially, MH questions were used to certify the performance of the teams as coordinators of care, as well as integration and resolubility actions. However, this set of items did not make it possible to distinguish, from the IRT, the teams regarding their level of coordination. Thus, through a research group consensus, 35 items of the questionnaire were selected, in addition to the conception adopted by the MH.

All items were recoded into ordered response categories from the worst-case scenario to the best-case scenario, according to the context of each item. The items with multiple answers were categorized considering the frequency of responses by the teams and the ordering in scenarios. Eight items related to the exams requested by the team were grouped into a single item, with two categories of response: whether or not to request all exams. The category “does not know/did not respond” was considered as missing data.

The PMAQ-AB questionnaire has dichotomic and polyatomic items. In this sense, the Item Response Theory model for graduated answers – Samejima’s Graded Response Model (GRM) – was used to obtain scores associated with the level of coordination^18^. The adjustment of the data to the GRM model was carried out in R, a statistical programming environment, through the ltm package^15^. Cronbach’s alpha and Spearman’s coefficient and the point-biserial correlation were used to analyze the internal consistency and the correlation between the items and between the items and the total score.

For each item analyzed, the response probability of the primary care teams for each item category was evaluated according to the level of coordination through the characteristic curves of the items. In this step, we evaluated the discrimination capacity of each item in the final composition of the measurement instrument. Items that were not able to differentiate teams regarding the level of coordination were removed.

To guarantee the model’s adequacy, two basic assumptions of the Item Response Theory were evaluated: the unidimensionality (the set of items should measure only one latent trait) and the local independence of the items (given a level of coordination, the teams’ responses for any item must be independent). The model’s unidimensionality was verified through principal component analysis. The presence of local independence is associated with unidimensionality^10^.

The proposed model allows the estimation of the discrimination parameters (a) of an item and the location (b) of the response categories. The estimation of the parameters in the Item Response Theory is known as calibration and generally uses the maximum likelihood method through the application of computational iterative processes. The discrimination parameter allows us to investigate the item’s ability to distinguish teams regarding the level of coordination. In practice, the discrimination parameter assumes values between zero and three, not admitting negative values. In this model, the lease parameter of a category corresponds to the coordination level of a team for which the probability of choosing the top category is 50%. The values of this parameter are expressed on the same scale as the scores (-3 a +3)^3^.

With the adjustment of the model, we obtained the estimates of the items’ parameters and the score associated with the level of coordination of each team. Subsequently, we performed a descriptive analysis of the scores, considering the distribution of frequencies, measures of central tendency, and dispersion. The score scale was maintained on the usual scale of the Item Response Theory (-3 to +3) and divided into degrees of coordination levels.

Small texts were associated with the answers of the item categories, organized in word cloud graphs. This resource helped, from the distribution of response frequencies to the items, in the interpretation of the obtained scores and visualization of their relationship with the level of coordination characteristics in which a certain team is located.

The study complies with the research guidelines involving humans established in Resolution 196/96 and was approved by the Research Ethics Committee of the Universidade Federal de Minas Gerais, on May 30, 2012, record 28804.

## RESULTS

Some items of the questionnaire initially chosen for the present study were excluded after preliminary analysis because they presented little variability in the response pattern for teams with distinct levels of coordination. Thus, they did not contribute to the achievement of the interest measure, because they had low or no capacity for discrimination. Thus, we applied the procedure of response categories recoding, when applicable, or the exclusion of items with problems in discrimination capacity.

The items removed were: standard template for filling in medical records; description of the diagnosis/problem/condition hypothesis in the medical record; description of the examinations requested in the medical record; electronic record implanted in the team; service available for user removal; scheduling appointments and actions for users who need continued care; forms for the other points of attention.

Seventeen items with two response categories and five with three response categories remained. The items and their ratios for each response category are shown in Table 1. The items on participation/use of telehealth and electronic medical record integrated with other points of the network had a greater proportion of responses in the worst-case scenario. On the other hand, the presence of a consultative appointment center, the sharing of the agenda by the team, the request for tests for the main conditions monitored by the APS, and the survey/mapping of the need for home care presented a higher proportion of responses from the best-case scenario.

**Table 1:** Distribution of the proportion of each response category. Brazil, 2012.

Item	Response categories proportion (%)			

	1	2	3	Missing data
Telehealth participation^a^	75.4	23.6	n.a.	0.9
Telehelth usage^a^	80.4	18.7	n.a.	0.9
Clinical qualification^a^	53.0	46.9	n.a.	0.1
Matrix support actions^b^	14.7	41.3	43.1	0.9
NASF support^a^	42.5	56.0	n.a.	1.5
CAPS support^a^	57.3	41.8	n.a.	0.9
Specialists support^c^	32.0	39.2	27.3	1.5
Integrated electronic medical record^a^	88.9	11.0	n.a.	0.1
Schedule shared by the team^a^	22.3	77.3	n.a.	0.4
Types of referral^d^	32.6	67.3	n.a.	0.1
Registration of higher risk users^a^	53.1	46.3	n.a.	0.6
Therapeutic protocols^e^	38.4	61.6	n.a.	-
Request for exams^f^	28.8	71.2	n.a.	-
Scheduling center^a^	9.3	90.7	n.a.	0
Defined references and flows^a^	42.5	55.5	n.a.	2.0
Exchange of information EqAB/Specialists^g^	33.1	52.3	14.6	0
Exchange of information Specialists/EqAB^g^	51.8	41.7	6.5	0
Institutional communication flow^h^	52.1	39.9	8.0	-
Telephone/Internet^a^	59.1	40.9	n.a.	-
Specialists contact list^a^	57.0	43.0	n.a.	0
Active search in the territory^i^	43.1	56.9	n.a.	0
Home care survey/mapping^a^	29.6	70.4	n.a.	-

n.a.: not applicable; NASF: *Núcleo de Apoio à Saúde da Família* (Family Health Support Center); CAPS: *Centro de Atenção Psicossocial* (Psychosocial Care Center); EqAB: Primary care team

^a^ 1 - No; 2 - Yes.

^b^ 1 - No action; 2 - From 1 to 5 actions; 3 - Above 6 actions.

^c^ 1 - Receives no support; 2 - Support from 1 to 10 CBO (Brazillian Occupation Code); 3 - Support from more than 10 CBO.

^d^ 1 - User tries to schedule an appointment and/or there is no defined path; 2 - Patient leaves the Unit with a scheduled appointment and/or the appointment is scheduled by the Unit, with later notice to the user.

^e^ 1 - It has less than 7 protocols; 2 - It has 7 or more protocols.

^f^ 1 - Requests all exams for less than 7 health conditions; 2 - Request all the exams for 7 to 8 health conditions.

^g^ 1 - Never; 2 - Yes, sometimes; 3 - Always.

^h^ 1 - There is no flow; 2 - There are 1 to 3 flows; 3 - There are more than 4 flows.

^i^ 1 - Performs in up to 6 cases (symptomatic respiratory, failing and monitored women); 2 - Performs in all cases.

Most primary care teams received more than six matrix support actions (43.1%), the highest response category of the item. In a different way, less than 10% of the teams responded to the highest category for the frequent exchange of information between the network and primary care professionals and for the existence of more than four institutional communication flows.

Table 2 shows the estimated values of the discrimination and location parameters of the items, as well as the values of internal consistency with the item exclusion and the correlation of each item with the total score. Regarding the internal consistency of all items, the general Cronbach’s alpha was 0.8018, which is not improved with the exclusion of any item. The correlation of the items with the final grade ranged from 0.258 (item 13) to 0.767 (item 18). Although they presented a low correlation with the total score, items 8, 9, 13, and 14 were maintained in the model due to their importance in the concept of coordination and its use by the MH.

**Table 2:** Estimates of the items parameters, internal consistency, and correlation with the total score. Brazil, 2012.

Item	Parameter (SD)^a^			Internal consistency^b^	Correlation with the total score
	
	a	b1	b2	Total (0.8018)	
1. Telehealth participation	0.807 (0.025)	1.630 (0.047)	n.a.	0.797	0.328
2. Telehealth usage	0.839 (0.027)	1.979 (0.056)	n.a.	0.798	0.313
3. Clinical qualification in team meeting	1.108 (0.026)	0.139 (0.017)	n.a.	0.790	0.483
4. Matrix support actions	1.266 (0.025)	-1.753 (0.032)	0.276 (0.015)	0.784	0.568
5. NASF support	0.752 (0.022)	-0.413 (0.025)	n.a.	0.795	0.353
6. CAPS support	0.814 (0.022)	0.446 (0.024)	n.a.	0.794	0.377
7. Specialist support	0.766 (0.019)	-1.091 (0.033)	1.390 (0.046)	0.797	0.391
8. Integrated electronic medical record	0.905 (0.032)	2.632 (0.078)	n.a.	0.800	0.271
9. Schedule shared by the team	0.588 (0.023)	-2.264 (0.083)	n.a.	0.801	0.246
10. Types of referral	0.832 (0.023)	-0.995 (0.031)	n.a.	0.797	0.371
11. Registration of higher risk users	0.844 (0.022)	0.192 (0.021)	n.a.	0.794	0.395
12. Therapeutic protocols	1.011 (0.025)	-0.567 (0.021)	n.a.	0.793	0.445
13. Request for exams	0.569 (0.021)	-1.703 (0.063)	n.a.	0.800	0.258
14. Scheduling center	0.900 (0.034)	-2.872 (0.090)	n.a.	0.800	0.259
15. Defined references and flows	1.202 (0.027)	-0.276 (0.017)	n.a.	0.790	0.510
16. Exchange of information EqAB/Specialists	0.989 (0.021)	-0.833 (0.024)	2.125 (0.086)	0.792	0.485
17. Exchange of information Specialists/EqAB	1.040 (0.023)	0.089 (0.018)	2.988 (0.260)	0.791	0.486
18. Institutional communication flow	2.429 (0.058)	0.063 (0.012)	1.747 (0.502)	0.782	0.767
19. Telephone/Internet	2.618 (0.075)	0.276 (0.012)	n.a.	0.786	0.749
20. Specialists contact list	0.995 (0.024)	0.341 (0.020)	n.a.	0.793	0.448
21. Active search in territory	0.680 (0.021)	-0.450 (0.027)	n.a.	0.797	0.329
22. Home care survey/mapping	0.768 (0.023)	-1.267 (0.039)	n.a.	0.797	0.338

NASF: Núcleo de Apoio à Saúde da Família (Family Health Support Center); CAPS: Centro de Atenção Psicossocial (Psychosocial Care Center); EqAB: Primary Care Team, SD: standard deviation; n.a.: does not apply

^a^ “a” (discrimination); “b1” and “b2” (response categories parameters).

^b^ Internal consistency with itemexclusion

**Figure 1:**
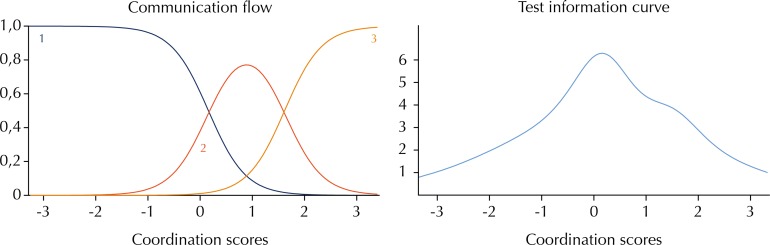
Characteristic curve of item 18 and test information curve.

The existence of a communication channel (telephone/Internet) and the number of institutional communication flows (case discussion, technical meetings with specialists, telehealth, teleconference, electronic medical record, reference/counter reference file, and electronic communication) showed lesser discrimination power, with discrimination parameters of 2.618 and 2.429, respectively. The characteristic curves of the communication flow items (item 18) and the information curve of the test can be seen in Figure 1, indicating a good behavior for item 18 to discriminate the teams regarding their level of coordination and the median region of the scale as the area of highest information accuracy for the coordination level.

Line 1: has no institutional communication flow.

Line 2: has one to three institutional communication flows.

Line 3: has more than four institutional communication flows.

Regarding the location of the item, two items had a greater value in the coordination level scale: the response category “always” to the frequency with which the network specialists contact primary care professional for information exchange, followed by the category “yes” for the presence of an electronic medical record integrated to the other points of the network. This means that such response categories were very likely to be present in teams with a higher level of coordination.

In the analysis of main components, the strong decrease in the percentage of variability between the first (28.4%) and second (8.3%) components indicated that the assumption of unidimensionality can be considered valid.

Once the item and skills parameters were estimated in the same metric, the coordination level scores were obtained, with values between -2.67 (minimum) and 2.83 (maximum). The mean was 0.0095 and the median was 0.0029. The score scale was divided into four levels (Table 3): well above average (scores greater than 1.5); above average (greater than zero and less than or equal to 1.5); below average (greater than -1.5 and less than or equal to zero); and well below average (less than or equal to -1.5).

**Table 3:** Distribution of primary care teams according to coordination scores ranges. Brazil, 2012.

Level (scores)	Absolute frequency (n)	Relative frequency (%)
Well below average (-3 to -1.5)	727	4.2
Below average (-1.5 to 0.0)	7,849	45.6
Above average (0.0 to 1.5)	7,791	45.3
Well above average (1.5 to 3.0)	835	4.9

Total	17,202	100

**Figure 2:**
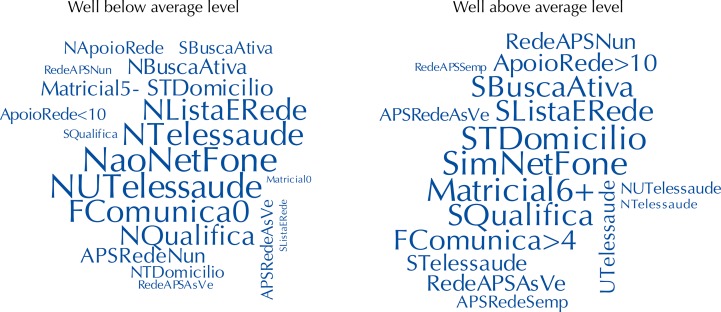
Word cloud: visualization of the response categories frequencies at the “well below average” (left) and “well above average” levels (right).

Figure 2 shows a cloud-type chart of the frequency distribution of response categories in two groups of teams with distinct levels of care coordination. On the left, the teams with the worst (4.2%) results and, on the right, the teams with the best (4.9%) results. At the “well below average” level, teams generally opted for lower response categories. As an example, they showed a greater probability of non-participation and non-use of telehealth, as well as a greater probability of not having institutional communication flows and the non-existence of telephone/Internet.

NListaERede: do not have a specialist contact list; SListaERede: have a specialist contact list; APSRedeNUN: there is never contact between primary care professionals and specialists to exchange information; APSRedeAsVe: sometimes there is contact between primary care professionals and specialists to exchange information; APSRedeSemp: there is always contact between primary care professionals and specialists to exchange information; RedeAPSNun: there is never a contact between specialists and professionals in primary care to exchange information; RedeAPSAsVe: sometimes there is contact between specialists and primary care professionals to exchange information; RedeAPSSemp: there is always contact between specialists and primary care professionals to exchange information; FComunica>4: more than four institutional communication flows; FComunica0: no institutional communication flow; NaoNetFone: no communication channel (telephone/Internet); SimNetFone: has a communication channel (telephone/Internet); NTelessaúde: does not participate in Telehealth; STelessaúde: participates in Telehealth; NUTelessaúde: does not use Telehealth for second formative opinion, telediagnostic and teleconsulting; UTelessaúde: uses Telehealth for second formative opinion, telediagnostic and teleconsulting; Matricial0: does not receive matrix support actions; Matricial5-: receives 1 to 5 matrix support actions; Matricial6+: receives 6 or more matrix support actions; NBuscaAtiva: does not perform active search; SBuscaAtiva: performs active search; NTDomicílio: conducts survey and mapping of home care; STDomicílio: carries out survey and mapping of home care; ApoioRede<10: receive support from 1 to 10 different professional occupation specialists; ApoioRede>10: receive support from more than 10 different professional occupation specialists; NQualifica: do not perform clinical qualification in team meetings; SQualifica: perform clinical qualification in team meetings

On the other hand, the categories of highest item response were more frequent among teams that fit the “well above average” level. These teams were more likely to present domains such as communication channel presence, a greater number of institutional communication flows, matrix support actions and frequent contacts between primary care professionals and specialists for information exchange.

We found that responses related to more communication, information exchange, matrix support, care in the territory and domicile had a significant weight in the teams with higher levels of coordination. This way, the simultaneous presence of these attributes to a greater degree can be considered a characteristic of the teams with higher positioning in the coordination level scale.

## DISCUSSION

The study showed the quality of the PMAQ-AB items related to the coordination and an assessment of the coordination level of the Brazilian primary care teams. The availability and transfer of information, the matrix support and the health care in the territory and the domicile were configured as elements of greater importance in the care coordination provided by the teams. Each participating team received a coordination score and, according to their performance, took a position at one of four levels of coordination created.

The importance of constructing a scale interpretable through the Item Response Theory made it possible to highlight the difficulties and facilities for care coordination in the PMAQ-AB, which shows great utility for the development and implementation of strategies according to the profile of each team.

The parameters’ invariance is another advantage of the Item Response Theory since it allows measurements that do not depend on the group nor on the instrument, since they are centered on the inference and properties of each item. This characteristic brings greater validity to the obtained scores and can be analyzed over time, allowing the monitoring of the advances achieved by the teams^17^.

It is worth mentioning that the issues evaluated in the present study were approached with a focus beyond the conception proposed by the MH. Other issues linked to health care were added. We considered the context of the teams, the context of transition between professionals and services and the context of the individual and community^7^. The broadening of the interventions context can bring up relevant information on care coordination. The elements must be analyzed from the dynamic and complex view of the nature of health services.

The elements of communication, matrix support actions, references and defined flows, the frequency of contact between specialists and primary care professionals, and the therapeutic protocols were the items with the greatest capacity to discriminate the coordination and had the highest correlation with the total score. Among the items considered more “difficult”, i.e., that required a higher level of coordination by the teams, are the frequency of contact between specialists and primary care professionals, the integrated electronic medical record, the use and participation in telehealth, support of network specialists and institutional communication flows. This finding, to a considerable extent, shows the importance of recognizing the needs of individuals and their previous care experiences for continued and timely care^21^.

The number of institutional communication flows, as the item with the highest correlation with the total score, indicates how the care provided by the teams depends on other spheres that are not strictly linked to the practices and processes of primary care. In this sense, health managers have the fundamental role of providing the structure necessary for care relationships to occur effectively^23^.

However, for quality relationships, the building of trust spaces for the appropriation of common goals depends on frequent contacts for the exchange of information between services and professionals. The frequent exchange of information between specialists and primary care professionals were items that required a higher level of coordination by the teams.

These relationships, when institutionalized, reflect a greater ability to coordinate care, insofar as they create important links in the formation of integrated networks. The present study showed that teams with a higher level of coordination opt for positive responses to issues that consider proximity in the relationships between those involved in the health care.

The support received by the primary care teams from different professionals in the qualification of the work process was another strong point. Teams that occupied the highest level of the scale are more likely to receive a greater number of matrix support actions, indicating the importance of strengthening the horizontal relationships in the teams’ daily work^19^.

The contribution of continued education to improving the quality of care was also significant in PMAQ-AB. Among the teams with the best levels of coordination, a large part participates and uses telehealth as a second formative opinion, telediagnostic or teleconsulting. Fonseca Sobrinho et al.^9^ emphasized the role of continued education as a matrix support activity in increasing the chances of obtaining a better certification in PMAQ-AB.

The PMAQ-AB actions developed in the territory to guarantee continuity of care also presented a relevant weight in the levels of coordination. Home care and the active search for individuals who require continuous monitoring had a significant frequency in the four levels constructed. This finding was corroborated by an Australian study^4^, which verified the success (or failure) of the coordination associated mostly with the professional response capacity at the local level, rather than the structural factors related to the availability and transfer of information from the various levels of the system.

In this sense, the monitoring of individuals over time is the task of the primary care teams through constant monitoring of different health conditions. In addition, in order to better monitor its assigned population, the team needs to know its territory in order to plan the actions appropriately.

Some limitations were found in the present study. Firstly, the attention paid to the generalization of results, since the number of teams participating in the first cycle of PMAQ-AB was controlled and the adhesion was voluntary, which may have led to the selection of teams more committed to the work process. The linkage of team certification to refinancing may have created biases in responses. Second, the lack in the literature of a consensual definition for a better understanding of the coordination attribute. Third, the formatting of the PMAQ-AB questions made it difficult to sort out the categories of responses, although they have met the theoretical assumptions of the IRT model.

The study allowed us to understand the care coordination situation of primary care teams, indicating the elements of the Program that most discriminate the coordination, as well as those that require a greater level of coordination by the teams. Knowledge of the level of care coordination can be of immense value for the planning and organization of services. In this sense, the ability to provide information and the frequency of contact among professionals are important elements for comprehensive, continuous and high-quality care.

## Footnotes

a McDonald KM, Schultz E, Albin L, Pineda N, Lonhart J, Sundaram V, et al. Care Coordination Measures Atlas: updated Jun 2014. Rockville: Agency for Healthcare Research and Quality; 2014 [cited 2017 Apr 13]. (AHRQ Publication No. 14-0037- EF). Available from: https://www.ahrq.gov/sites/default/files/publications/files/ccm_atlas.pdf

b Ministério da Saúde (BR). Portaria Nº 1.654, de 19 de julho de 2011. Institui, no âmbito do Sistema Único de Saúde, o Programa Nacional de Melhoria do Acesso e da Qualidade da Atenção Básica (PMAQ-AB) e o Incentivo Financeiro do PMAQ-AB, denominado Componente de Qualidade do Piso de Atenção Básica Variável - PAB Variável. Brasília (DF); 2011 [cited 2017 Apr 13]. Available from: http://bvsms.saude.gov.br/bvs/saudelegis/gm/2011/prt1654_19_07_2011.html
